# Association between dental agenesis and delay in dental development: a preliminary study in a Spanish paediatric population in relation with Dental Anomaly Pattern (DAP)

**DOI:** 10.1186/s12903-022-02522-6

**Published:** 2022-11-05

**Authors:** Carmen León-Rubio, Andrea Martín-Vacas, Gloria Saavedra-Marbán, Marta Macarena Paz-Cortés

**Affiliations:** 1 Faculty of Dentistry, Alfonso X El Sabio University, Avenida de La Universidad 1, 28691 Villanueva de La Cañada, Spain; 2 Department of Dental Clinical Specialties, Faculty of Dentistry, Complutense University of Madrid, Plaza Ramón y Cajal s/n, 28040 Madrid, Spain

**Keywords:** Dental anomaly pattern, DAP, Dental agenesis, Dental development, Paediatric dentistry, Children

## Abstract

**Background:**

The association between dental anomalies has been studied, giving rise to the concept of Dental Anomaly Pattern (DAP). Tooth agenesis has been associated with alterations such as molar infracclusion, taurodontism and delayed dental development. The aim of this study was to evaluate the dental development pattern in patients with non-syndromic dental agenesis, in comparison with a control group.

**Methods:**

Dental and chronological age was analysed in a sample size of 204 orthopantomographs divided into a study group (*n* = 104) and a control group (*n* = 100) with the Demirjian Method. Intra and intergroup differences in chronological and dental age, and the correlation between them were calculated by statistical analysis with a 95% confidence level (*p* < 0.05).

**Results:**

Dental age exceeded chronological age both in the control group and in the study group. Statistically significant differences (*p* = 0.004) were found when comparing the difference between chronological and dental age in the study (-0.16 ± 1.12) and control group (-0.58 ± 0.90). Regarding sex and age intergroup differences, the results were only statistically significant in the girls’ group (*p* = 0.017), and the age over 8 years old (*p* < 0.05). There were no significant differences in tooth development depending on the number of missing teeth or the affected tooth group, but there was a delay in the development of the homologous tooth contralateral to the absent one in 14.9% of patients.

**Conclusions:**

The difference between chronological and dental age in permanent dentition is significantly lower in Spanish children with non-syndromic agenesis compared to a control group, presenting a lower dental age than chronological age than children without non-syndromic agenesis.

## Background

In the early 1960s, Gran et al. established the possibility that certain polymorphisms in the number of teeth were not isolated phenomena, but that they were fundamentally related to other dental anomalies of size, development, and eruption [1]. In the 1990s, Sheldon Peck's group studied associations between dental anomalies [2–8]; giving rise to the definition of the concept of Dental Anomalies Patterns (DAP), which includes a set of dental abnormalities that appear associated with much more frequency than is explained at random [9]. The nine anomalies included in DAP are dental agenesis, localized microdontia, generalized or localized tooth reduction, delayed formation and localized or generalized tooth eruption, dental infraocclusion, displacement of the maxillary permanent canine towards the palate, two types of transpositions and distal angulation of the non-erupted lower second premolar [4, 9, 10].

Due to the high prevalence of non-syndromic dental agenesis, which can affect 25% of the general population, it is one of the most studied anomalies [11]. In the recent literature, it can be found studies analysing the relationship between dental agenesis and other dental anomalies such as microdontia, delay in tooth development, eruptive alterations, transpositions, taurodontism and alterations in tooth angulation or position [12, 13]. Recent genetic studies have found an association between dental agenesis and mutations in multiple genes, so the hypothesis that there is an aetiology based on multiple genes and protein products involved in dental agenesis and these anomalies supports the theory of DAP, with great intra- and intersubject variability in phenotypic manifestation, even within the same family [14].

Recent studies maintain that certain genes associated with the absence of dental germ could also be the cause of the delay in the development of present teeth. There is currently no agreement on the association between tooth agenesis and delayed dental development [15–18]; however, the importance of permanent dental development and the chronology of dental eruption are of great relevance in the planning of treatments in paediatric dentistry and orthodontics. The aims of this study were to evaluate the dental development pattern in patients with non-syndromic dental agenesis compared to a control group, and to study localized dental development in patients with unilateral dental agenesis compared to the contralateral side.

## Material and methods

### Study design

This study was designed as a cross-sectional observational study and it is reported following the STROBE guidelines. In accordance with current regulations, the current legal framework regarding Personal Data Protection was respected throughout the study process, coding the patients included in the study to maintain their anonymity and the study blinded. In addition, the study was approved by the Ethical Committee for Clinical Research of the “Clinico San Carlos Hospital” (reference 19/444-E) and considers all aspects of the Helsinki Declaration. Before conducting the study, the patients or their legal representatives signed the informed consent to be part of the study.

### Patient sample

The study population consisted of patients who had attended a private radiodiagnosis clinic in Madrid, Spain, during the last decade, having demographic data and an orthopantomography of the initial diagnosis and subsequent control. Sample size was calculated for the total sample using the GRANMO tool based on the results obtained by Tunç et al. [19] accepting an alpha risk of 0.05 and a beta risk of 0.2 in a bilateral contrast, and assuming a common standard deviation of 0.76; 101 subjects were required in the experimental group and 101 in the control group to detect a difference equal to or greater than 0.3 units. A loss to follow-up rate of 0% was estimated. Applying the "agenesis" filter to the radiograph database, a population of 320 radiographs of patients with agenesis and the selected age range between 6 and 15 years of age was obtained. After applying systematic probabilistic sampling (interval = 3), 106 orthopantomographs were obtained. Applying the inclusion and exclusion criteria (Table 1), 2 samples were eliminated due to poor image quality, with a final sample of 104 orthopantomographs in the study group. The control sample was obtained from patients without a filter from the radiographic manager with the selected age range, with a probabilistic systematic sampling (interval = 3) until completing the 101 established in the calculation of the sample size. Subsequently, one radiograph had to be eliminated due to poor image quality. Patients with bilateral tooth agenesis of the premolars had to be excluded, since this condition prevents dental age assessment by the Demirjian method. Finally, 204 subjects aged between 6–15 years old were considered, 104 were part of the group with dental agenesis (46 boys and 58 girls) and 100 from the control group (42 boys and 58 girls).

**Table 1:** Inclusion and exclusion criteria

Inclusion criteria	Exclusion criteria
- 6–15 years old - Dental agenesis - No orthodontic treatment - No craniofacial syndrome - No general disease or trauma that could affect dental development - Caucasian children	- Bilateral dental agenesis in the lower jaw - No subsequent records for the accurate diagnosis of tooth agenesis - Poor radiography quality

### Study procedure and methods

For the diagnosis of dental agenesis, as well as the determination of dental development and maturation, orthopantomographs were used, performed with the same Orthoceph® OC 100D machine. Image quality criteria were assessed, such as radiographic artifacts (jewels or appliances), patient mobility during the radiography, image overlap, etc. All those distorted images were excluded according to Table 1. Since they were all made with the same equipment, exposure, magnification, and resolution were the same in all records.

Analysis of the radiographs was carried out by two examiners under the same observation conditions. Both examiners are trained and calibrated paediatric dentists with extensive experience in determining age with the Demirjian method. After 1 month, the operators were asked to re-evaluate a 25% of the sample and the Kappa Cohen coefficient was calculated to assess the degree of agreement between the examiners.

A non-erupted tooth was considered a diagnosis of tooth agenesis, the germ of which does not appear in the orthopantomography and has not been extracted or accidentally lost. To confirm the certainty of dental agenesis, in included patients younger than 10 years a follow-up radiograph was required after 10 years of age to rule out late dental development.

The determination of the developmental stage was carried out with the Demirjian method [20, 21], evaluating the seven left mandibular teeth from distal to mesial. In case of dental agenesis of the left premolar or premolars, the contralateral right counterparts were evaluated. Using the author's conversion tables [20], the *dental age* was calculated. The *chronological age* of the patient was calculated with the date of birth of the patient and the date of the orthopantomography, measured in years. To determine the existence and quantify the delay in dental development, dental age was subtracted from chronological age.

The development of the contralateral homologous tooth was studied in those patients with unilateral dental agenesis, finding the difference between the “existing stage” in the orthopantomography and the “expected stage” according to the maturation tables proposed by Paz Cortes et al. [22]. The frequency and percentage of premolars contralateral to the absent one that were in the appropriate stage, or a stage higher or lower than expected for their age was assessed.

### Statistical analysis

Statistics were performed to analyse the differences between dental age (DA) and chronological age (CA) in the control group and the group with dental agenesis. The data were analysed with the SPSS version 15 program. The following statistical tests were performed with a confidence level of 95% (*p* < 0.05):

- Descriptive statistics for qualitative (frequency, percentage) and quantitative (mean, standard deviation, maximum, minimum, etc.) variables.
- Kolmogorov–Smirnov test to analyse the normality of the sample.
- Fisher test or Chi square test, to contrast the independence or influence between two qualitative variables.
- Parametric Student's t test for the comparison of two means (difference between DA and CA) in quantitative variables, when normality in the data is assumed. The equality of variances is contrasted with the Levene test.
- Non-parametric Mann–Whitney and Wilcoxon test to compare the mean of a quantitative variable (difference between DA and CA) between two groups, when normality in the data is not assumed.
- ANOVA test and Bonferroni test for the comparison of more than two independent variables, when normality in the data is assumed.
- Spearman’s Rank correlation test to assess the chronological age-dental age correlation.

## Results

Two hundred four orthopantomographs of paediatric patients were analysed. Intra and inter-operator concordance was measured prior to analysis of the study results. Both intraoperator (K Cohen value 0.980) and interoperator concordance (K Cohen value 0.991) were considered excellent, so the research is considered to be reliable and reproducible.

51% of the total sample (104 patients) had dental agenesis of at least one permanent tooth, the remaining 49% (100 patients) constituting the control group. In boys the prevalence of tooth agenesis was 52.3%, and in girls 50%, without statistically significant differences (*p* = 0.937) (Tables 2 and 3).

**Table 2:** Prevalence of tooth with agenesis

	Tooth	Maxilla		Mandibula	
		N	Percentage	N	Percentage
RIGHT HEMIARCADE	2^nd^ molar	0	0%	1	0.96%
	1^st^ molar	0	0%	0	0%
	2^nd^ premolar	6	5.7%	11	10.57%
	1^st^ premolar	1	0.96%	0	0%
	Canine	1	0.96%	0	0%
	Lateral incisor	57	54.8%	2	1.92%
	Central incisor	0	0%	1	0.96%
LEFT HEMIARCADE	Central incisor	0	0%	4	3.84%
	Lateral incisor	50	48.07%	1	0.96%
	Canine	1	0.96%	0	0%
	1^st^ premolar	1	0.96%	0	0%
	2^nd^ premolar	5	4.8%	13	12.5%
	1^st^ molar	0	0%	0	0%
	2^nd^ molar	0	0%	0	0%

**Table 3:** Distribution of the amount of dental agenesis per patient and sex

Sex	Dental Agenesis (number)	Frequency (percentage)		
		Boys	Girls	TOTAL
Total	1	26 (56.5%)	33 (56.9%)	59 (56.7%)
	2	18 (39.1%)	22 (37.9%)	40 (38.5%)
	3	2 (4.3%)	1 (1.7%)	3 (2.9%)
	4	0 (0%)	2 (3.4%)	2 (1.9%)
	Total	46 (100%)	58 (100%)	104 (100%)

The mean chronological age for both sexes in the agenesia group was 10.0yrs ± 2.0 (10.2yrs ± 2.1 in boys, and 9.8yrs ± 1.0 in girls). The mean chronological age in the control group was 8.5yrs ± 1.2, being 8.8yrs ± 1.3 in boys and 8.8yrs ± 1.2 in girls. Statistically significant differences were found in chronological age in the two study groups both in the total sample (*p* < 0.001) and by sex (*p* = 0.002).

The mean dental age for both sexes in the group with dental agenesis was 10.1yrs ± 2.3 (10.4yrs ± 2.5 in boys and 9.9yrs ± 2.2 in girls). In the control group, the mean dental age for both sexes was 9.4yrs ± 1.7 (9.3yrs ± 1.6 in boys and 9.4yrs ± 1.7 in girls). No statistically significant differences were found in the total sample (*p* = 0.058) or in girls (*p* = 0.425). Differences between boys in the group with dental agenesis and the control group were statistically significant (*p* = 0.047).

**Fig. 1:**
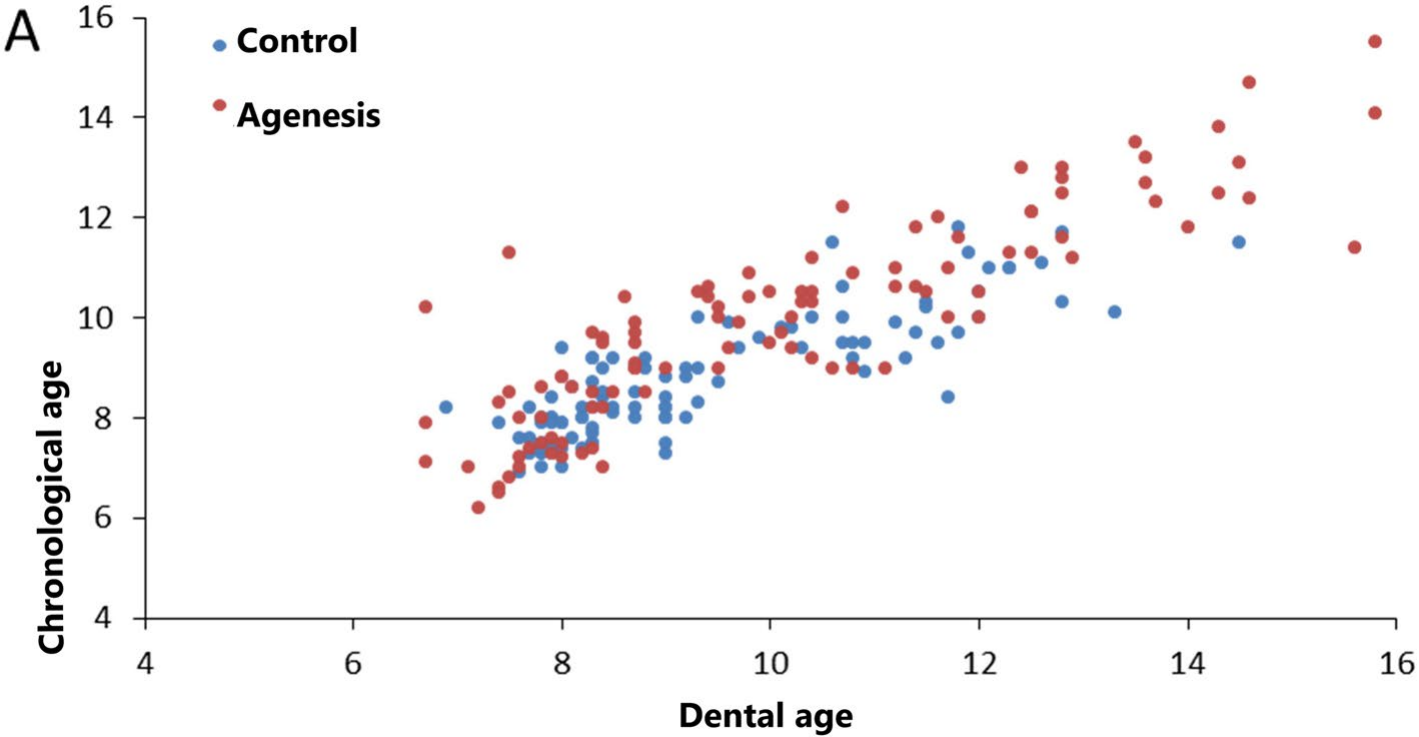
Correlation of dental age and chronological age

A positive correlation was found between chronological and dental age (correlation coefficient 0.848, *p* < 0.001). The correlation coefficient in the group with tooth agenesis was 0.876 and in the control group 0.837 (Fig. 1).

In the group with dental agenesis, the difference between chronological and dental age was -0.16yrs (± 1.12) while in the control group, the value was -0.58yrs (± 0.90). Statistically significant differences were observed between the group with dental agenesis and the control group (*p* = 0.004) (Table 4). The difference found between girls in the group with tooth agenesis with respect to those in the control group was statistically significant (*p* = 0.017), unlike that found between boys in the group with dental agenesis with respect to the control group (*p* = 0.105). No statistically significant differences were found in the intragroup analysis by sex, neither in the group with tooth agenesis (*p* = 0.93) nor in the control (*p* = 0.61).

**Table 4:** Mean difference between chronological and dental age, and statistical significance (p)

	CA^a^ – DA^b^ mean (SD^c^)			Mean difference between the two groups for the total sample			Significance level (p) for differences (CA-DA) between the two groups		
	Total (yrs)	Boys (yrs)	Girls (yrs)	Mean (yrs)	Upper limit (95% CI^d^)	Lower limit (95% CI)	Total	Boys	Girls
Dental Agenesis group	-0.16 (± 1.12)	-0.18 (± 1.01)	-0.14 (± 1.21)	0.42	0.138	0.702	0.004	0.105	0.017
Control group	-0.58 (± 0.90)	-0,49 (± 0,70)	-0.65 (± 1.02)						

^a^*CA* Chronological age

^b^*DA* Dental age

^c^*SD* Standard Deviation

^d^*CI* Confidence Interval

Patients were grouped into three categories according to their age (under 8, between 8 and 10, and over 10 years). The differences between chronological and dental age within each age group were analysed. Intragroup differences were studied and, while in the group with dental agenesis, no significant differences were found (*p* = 0.434), statistically differences were found in the control group (*p* = 0.007). The differences in the control group were found between the group with age less than or equal to 8 years of age and those older than 10 years (*p* = 0.015), in addition to between children between 8 and 10 years and those older than 10 years (*p* = 0.007) (Table 5).

**Table 5:** Mean difference between chronological and dental age in chronological age subgroups, and statistical signification (p)

CA^a^ group (n)	Intragroup comparison (CA-DA^b^)		Intragroup comparison (CA-DA) between the CA groups				
	Mean (± SD^c^) (yrs)	p	CA group	Mean (yrs)	Significance level (p)	CI^d^ (95%)	
						Upper limit	Lower limit
	DENTAL AGENESIS						
≤ 8 (*n* = 20)	-0.41 (± 0.6)	0.434	8–10	-0.40929	0.594	-1.1785	0.36
			> 10	-0.24153	1	-0.9697	0.4867
> 8–10 (*n* = 35)	-0.005 (± 0.97)		≤ 8	0.40929	0.594	-0.36	1.1785
			> 10	0.16776	1	-0.4396	0.7751
> 10 (*n* = 49)	-0.17 (± 1.36)		≤ 8	0.24153	1	-0.4867	0.9697
			8–10	-0.16776	1	-0.7751	0.4396
	CONTROL						
≤ 8 (*n* = 35)	-0.48 (± 0.48)	0.007	8–10	-0.03371	1	-0.4978	0.4304
			> 10	0.76762	0.015	0.1177	1.4175
> 8–10 (*n* = 50)	-0.45 (± 0.98)		≤ 8	0.03371	1	-0.4304	0.4978
			> 10	0.80133	0.007	0.1814	1.4213
> 10 (*n* = 15)	-1.25 (± 1.08)		≤ 8	-0.76762	0.015	-1.4175	-0.1177
			8–10	-0.80133	0.007	-1.4213	-0.1814

^a^*CA* Chronological age

^b^*DA* Dental age

^c^*SD* Standard Deviation

^d^*CI* Confidence Interval

When studying the intergroup difference between the study and control groups between chronological and dental age, depending on the age group, statistically significant differences were found in the group of patients between 8 and 10 years of age (*p* = 0.043) and in the group over 10 years of age (*p* = 0.007) between the group with dental agenesis and the control group.

To analyse the differences between chronological and dental age as a function of the amount of dental agenesis per subject, subjects with single agenesis and agenesis of two or more teeth were analysed. A difference of 0.29 years was observed between both groups, without statistically significant differences (*p* = 0.188). Differences were also analysed according to the affected dental group, dividing the sample into incisor agenesis (*n* = 73) and premolar agenesis (*n* = 28); the difference in years between the chronological and dental ages between both groups was 0.24 years, with no statistically significant differences (*p* = 0.338).

The development of the contralateral homologous tooth was studied in patients with unilateral dental agenesis (*n* = 54). 64.8% of the patients were in the expected stage for their age, while 14.9% of the patients were in stages below those expected and one subject (1.9%) was ahead of the expected stage.

## Discussion

A discussion was carried out on both the methodology and results of our study, as well as the comparison of existing studies in the literature (Table 6) [17, 19, 23–28].

**Table 6:** Previous studies of association between dental agenesis and delay in dental development [17, 19, 23–28]

Author	Year	Population	Sample size	Age	Dental Agenesis (amount)	Method	Delay (years)
Rune et al. [27]	1974	Sweden	91	7–19	6	Haavikko	1.8 (boys) 2.0 (girls)
Odagami et al. [28]	1995	Japan	1623	5–10		Moorrees	NO
Uslenghi et al. [24]	2006	United Kingdom	135	3–15	1–2	Haavikko	1.49 (girls) 1.53 (boys)
Kan et al. [25]	2010	Australia	230	5–15	1–4	Demirjian	0.9 (boys) 1.1 (girls)
Tunç et al. [19]	2011	Turkey	210	5–12	1–5	Demirjian	0.3 (boys) 0.3 (girls)
Ruiz-Mealin et al. [17]	2012	United Kingdom	139	9–17,6	1–6	Demirjian	0.84 (boys) 0.88 (girls)
Ruiz-Mealin et al. [17]	2012	United Kingdom	139	9–17,6	1–6	Haavikko	0.9 (boys) 0.6 (girls)
Medina et al. [26]	2016	Venezuela	188	5–12	1–2	Nolla	NO
Badrov et al. [23]	2016	Croatia	345	6–15	1–2	Haavikko	0.57 (boys) 0.61 (girls)
Present study	2021	Spain	204	6–15	1–4	Demirjian	0.30 (boys) 0.50 (girls)

Analysing the sex distribution of our study sample, it has been ruled out as a possible confounding factor, since no statistically significant differences were found in either of the two study groups. Regarding the sample size (*n* = 204), it was similar to that of Tunç et al. [19], Ruiz-Mealin et al. [17], Uslenghi et al. [24], Kan et al. [25] and Medina et al. [26] and lower than Badrov et al. [23] and Odagami et al. [28]. The determination of the number of subjects in our study is considered adequate because it was based on a previous sample size calculation. The age of our sample ranged between 6 and 15 years of age, being similar to the studies by Park et al. [29], Tunç et al. [19], Medina et al. [26] and Odagami et al. [28]. Authors such as Garib et al. [8] admit the selection of a sample of various ethnicities as a limitation of the DAP study, so in our study we limited the sample to Caucasian race to limit possible ethnic bias.

The study sample had a significantly higher chronological age in the group with dental agenesis compared to the control. It has been observed in previous research that the Demirjian method [20, 21] offers differences depending on the population and the ethnic group analysed [17, 30–32], leading to an overestimation of dental age in Spanish population [22, 31, 33–35], so we determined that the difference between chronological and dental age is not influenced by the age of the subject. In both groups, the overestimation of the Demirjian method was confirmed, since the values ​​of the difference in chronological and dental age were negative, indicating a higher dental age than chronological age of the patients.

The difference in tooth development found in our study is statistically significant, finding a difference of 0.42 years (5 months) between the group with dental agenesis and the control group. In the group with tooth agenesis, the delay in dental development with respect to the control group was confirmed, with a significant difference in girls (0.5 years, 6 months) but not in boys (0.3 years, 3.6 months). The study by Tunç et al. [19], with a sample size similar to ours and using the Demirjian method, manifesting slightly lower differences between the ages studied, being 0.3 years in both sexes, with a significant delay in the group with dental agenesis. The results of similar studies [17, 23–25, 27] coincide with these results, with the greatest delay in dental development in the study by Rune et al. [27] with a difference of 1.8 years in children and 2 years in girls.

The influence of chronological age on dental development was studied. Significant differences were found between both groups in the subgroups of patients between 8–10 years and those older than 10 years, suggesting that as age increases so does the difference between chronological and dental age, as was observed by Uslenghi et al. [24]. Kan et al. [25] also found a greater delay in dental development in girls 9–10.9 years old and boys 11–12.9 years old, arguing that they coincide with periods of pubertal growth. Ruiz-Mealin et al. [17] even quantified that for each year of the patient, the delay in dental development increased 0.48 years.

Regarding the influence of the affected dental group, we only considered incisors (anterior group) and premolars, since the rest of the dental groups did not have enough patients to carry out a statistical study. We did not find significant differences in dental development between both groups, although the existence of a greater number of patients with dental agenesis of incisors over premolars (patients with bilateral dental agenesis of premolars had to be excluded) may have biased this analysis. Although most of the studies consulted consider agenesis without differentiating the dental group, Gelbrich et al. [36] concludes that the agenesis of the second premolar is not a purely local defect, since its association with the delay in dental maturation raises the suspicion of the existence of a common aetiology.

Our results indicate that in approximately 15% of the subjects, the dental development of the homologous tooth contralateral to the absent one is two or more stages lower than that expected for patients of the same age without dental agenesis. Navarro et al. [37] determined that the development of the contralateral premolars to the agenesis of mandibular second premolars present a delay of 0.5 years (6 months). Authors such as Uslenghi et al. [24] refer to a pattern of generalized delay in dental development, being more severe in the adjacent teeth, mesial and distal, to the site of dental agenesis. Ben-Bassat et al. [38] reached similar conclusions, reporting a delay in dental development with respect to its contralateral in 30.7% of teeth mesial to tooth agenesis, and in 10.2% of distal teeth. Daugaard et al. [39] stated that in subjects with dental agenesis changes in the maturation pattern are in the area affected by tooth agenesis.

Some limitations of the study need to be discussed. In the first place, the patients were not matched between the two groups studied, however, the variable analysed was the difference between the chronological and dental age of each subject, by which we minimize the role of age as a bias since no group means have been used. Because there are multiple factors that can alter body and dental development that have not been controlled due to the complexity of obtaining the data (for example, socioeconomic status or body mass index), the data must be interpreted for the general paediatric population but considering the individual characteristics of each child. On the other hand, the sample size was calculated for the selection of a child sample, without differentiating between age subgroups, therefore, interpretations within age groups must be considered with caution, since they are not matched. Likewise, it is understandable that fewer records are available in the group of younger children (6–8 years of age), since the request for orthopantomographs by dentists is restricted to therapeutic need, and in general, more orthodontic studies are carried out in children already in the second phase mixed dentition or permanent dentition.

Among the advantages of our study, we find that it is the first to use a Spanish sample of great importance due to the great variability in terms of development chronology and dental eruption in terms of race or cultural characteristics. In addition, there is no study from 2016 to date that relates dental versus chronological age and dental agenesis, so it is considered essential to carry out an update study to avoid the use of old data that may mislead, since it is It is known that evolutionary changes occur not only in body development, but also in terms of chronology and sequence of dental eruption.

Knowledge of dental and chronological age is basic not only in legal and forensic dentistry, but also in the daily planning of treatments in the dental office, especially orthodontic or orthopaedic treatments. It is important to consider dental agenesis and delay in dental development as part of DAP, both in the individualization of the patient's treatment plan and in future research on the epigenetic interaction that is involved in dental development. We agree it is necessary to establish future lines of research that carry out a multicentre study, with matching of subjects and control of confounding variables (body mass index, socioeconomic status, etc.) to establish a logistic regression analysis that allows the creation of a predictive model for the analysis of the interrelationship between dental and chronological age in patients with DAP.

## Conclusions

Within the limitations of the present study, it can be concluded that the difference between CA-DA in permanent dentition is significantly lower in Spanish children with non-syndromic agenesis (-0.16yrs ± 1.12) compared to a control group (-0.58yrs ± 0.90), presenting a lower DA than CA than children without non-syndromic agenesis. There is a delay in the development of the homologous tooth contralateral to the absent one in approximately 15% of patients.

## Data Availability

All collected data from patients analysed during this study are included in this published article.

## Abbreviations

CA: Chronological age
DA: Dental age
DAP: Dental Anomaly Pattern
Yrs: Years
